# Human Kidney Disease-causing INF2 Mutations Perturb Rho/Dia Signaling in the Glomerulus

**DOI:** 10.1016/j.ebiom.2014.11.009

**Published:** 2014-11-13

**Authors:** Hua Sun, Khaldoun I. Al-Romaih, Calum A. MacRae, Martin R. Pollak

**Affiliations:** a Nephrology Division, Department of Medicine, Beth Israel Deaconess Medical Center, Boston, MA 02215, United States; b Harvard Medical School, Boston, MA 02215, United States; c Cardiology Division, Department of Medicine, Brigham and Women's Hospital, Boston, MA 02115, United States; d Broad Institute of Harvard and MIT, Cambridge, MA, United States; e Iowa University Children's Hospital, Iowa City, IA 52242, United States

**Keywords:** INF2, Glomerulus, Rho

## Abstract

Mutations in Inverted Formin 2 (INF2), a diaphanous formin family protein that regulates actin cytoskeleton dynamics, cause focal segmental glomerulosclerosis (FSGS) and Charcot–Marie–Tooth Disease (CMT) in humans. In addition to directly remodeling actin filaments in vitro, we have shown that INF2 regulates intracellular actin dynamics and actin dependent cellular behavior by opposing Rhoa/Dia signaling. As a step towards a better understanding of the human kidney disease, we wanted to explore the relevance of these findings to the in vivo situation. We used dose dependent knockdown of INF2 to first define an in vivo model and establish an overt glomerular phenotype in zebrafish. This simple assay was validated by rescue with wild type INF2 confirming the specificity of the findings. The edema, podocyte dysfunction, and an altered glomerular filtration barrier observed in the zebrafish pronephros correlate with mistrafficking of glomerular slit diaphragm proteins, defective slit-diaphragm signaling, and disinhibited diaphanous formin (mDia) activity. In contrast to wild-type human INF2, INF2 mutants associated with kidney disease fail to rescue the zINF2 morphant phenotype. Of particular interest, this INF2 knockdown phenotype is also rescued by loss of either RhoA or Dia2. This simple assay allows the demonstration that INF2 functions, at least in part, to modulate Dia-mediated Rho signaling, and that disease causing mutations specifically impair this regulatory function. These data support a model in which disease-associated diaphanous inhibitory domain (DID) mutants in INF2 interfere with its binding to and inhibition of Dia, leading to uncontrolled Rho/Dia signaling and perturbed actin dynamics. Methods to fine tune Rho signaling in the glomerulus may lead to new approaches to therapy in humans.

## Highlights

• Dose dependent knockdown of zebrafish INF2 defines an overt glomerular phenotype.
• This phenotype is rescued by human wild-type INF2 but not by disease causing INF2 mutants.
• The developmental phenotype correlates with disinhibited diaphanous formin activity
• The INF2 knockdown phenotype is rescued by knockdown of either RhoA or Dia2
• INF2 mutations lead to uncontrolled Rho/Dia signaling and perturbed actin dynamics.

## 1 Introduction

Inverted formin 2 (INF2) is a member of the formin family of proteins that regulate actin dynamics (Chhabra and Higgs, 2006, Korobova et al., 2013, Ramabhadran et al., 2011). INF2 mutations cause kidney disease in humans characterized by focal segmental glomerulosclerosis (FSGS) with or without Charcot–Marie–Tooth disease (Brown et al., 2010, Barua et al., 2013, Boyer et al., 2011, Gbadegesin et al., 2012). Like other formins, INF2 is a multidomain protein whose function depends on the regulatory effect of the N-terminal Diaphanous Inhibitory Domain (DID) on the C-terminal Diaphanous Autoregulatory Domain (DAD). In addition to INF's direct actin polymerizing and depolymerizing activity, we have shown that INF2 also antagonizes Rho activated actin polymerizating activity of diaphanous related formin proteins (Dias) by the interaction of INF2-DID with Dia DAD (Chhabra and Higgs, 2006, Sun et al., 2011). Most of the disease-causing mutations occur in the DID, interfering with the INF2-DID/Dia-DAD interaction. In cultured cells, we have found that this prevents the dampening effect of INF2 on Rho/Dia signaling, leading to imbalanced actin dynamics, perturbation of actin polymerization, and disruption of actin based cellular remodeling processes (e.g. lamellipodia formation and trafficking to the membrane of cultured podocytes (Sun et al., 2013)).

In vivo podocytes are highly complex epithelial cells with projections, typically referred to as foot processes that make up the distal component of the glomerular filtration barrier. The slit diaphragm, a unique cell–cell junctional complex, is an electron dense structure visible between neighboring foot processes (Kubiak and Niemir, 2006, Asanuma and Mundel, 2003). Both the intricate morphology and unique function of podocytes are dependent on the regulation of the actin cytoskeleton, which is under the spatial and temporal control of the Rho family of small GTPases (Zhu et al., 2011, Scott et al., 2012, Akilesh et al., 2011, Mouawad et al., 2013, Oh et al., 2004, Shono et al., 2009). Uncontrolled activation of RhoA in RhoA transgenic mice has been shown to cause aggregation of actin bundles in podocytes, mislocation of slit diaphragm proteins, and proteinuria (Zhu et al., 2011, Gupta et al., 2013). Our in vitro findings indicate that INF2 maintains a balance in actin dynamics via control of Rho/Dia signaling mediated though the DID-DAD interaction. Here we extend our studies to the in vivo functional characterization of INF2 in the intact organism. We used a zebrafish model to investigate the in vivo role of INF2 and the mechanism by which INF2 participates in the pathophysiology of podocyte-mediated disease and, more generally, in nephrogenesis. We find that INF2 is required for normal zebrafish development and, specifically, for normal glomerular structure and function. We demonstrate that the function of INF2 in glomerular development is largely mediated by its effect on Rho-activated Dia signaling, as knockdown of either zebrafish Rhoa or Dia2 rescues the INF2 morphant phenotype. The relative simplicity of this in vivo model of INF2-associated disease and its rescue by transient transgenesis and gene knockdown support its utility in examining genetic and pharmacologic modulation.

## 2 Materials and Methods

### 2.1 Zebrafish, Morpholinos and PCR Primers

Experiments were performed on wild-type zebrafish (*Danio rerio*) in accordance with Institutional Animal Care and Use Committee (IACUC)-approved protocols. The INF2 translation blocking morpholino (INF2 ATG MO) and the zINF2 exon 3 splice (INF2 E3I3) morpholino were synthesized by Gene Tools, LLC (Philomath, OR), and titrated to 2–3 ng/embryo as the lowest concentration sufficient to induce abnormal phenotype. For each experimental group, at least 400 individual embryos were injected by using a micro injector PLI-90 (Harvard Apparatus, Cambridge, MA).

**Table:** 

Morpholinos	Sequence
zINF2 ATG MO	GGGCTCCCTCTGCCTTCATCGACAT
zINF2 E3I3 MO	AGAGTTAAGGTCACACTTGCCTTGT
Scrambled MO	CCTCTTACCTCAGTTACAATTTATA

Primers	Forward/reverse

zINF2Primer1	TAGGCTCGTCTCAGGACTC/GATCACACTGAAGCGATACTGC
zINF2 Primer 2	GCCCTCAGCATGTTCTCTTC/TCTTCAAACGCCTCACACTG
zINF2 Primer 3	AAGATTGGTTCCCTGCTGAA/TGATGGTGCTGAAGAGTTCG

### 2.2 In Situ Hybridization

PCR product 1 was cloned into pDrive Cloning Vector (QIAGEN PCR Cloning Kit). A digoxigenin-labeled riboprobe was generated by in vitro transcription with T7 (antisense) or SP6 (sense) RNA polymerase (Roche) from linearized constructs using Dig RNA labeling Mix (Roche). Whole-mount in situ hybridizations were performed as described (Thisse and Thisse, 2008).

### 2.3 In Vitro Transcription

Full-length human flag-tagged INF2 was subcloned from pCMV-flag-INF2 into the pxT7 vector between the KpnI and XhoI sites. The capped and tailed mRNA of wide type and mutants of hINF2 was transcribed from pxT7-hINF2 linearized with XbaI using the mMACHINE T7 Ultra Kit (Ambion, USA). Embryos were microinjected with 50 pg of wide type or mutated forms of human INF2 mRNA together with either control or INF2 morpholinos. The expression of INF2 in embryos injected with hINF2 mRNA was confirmed by Western blot using mouse-anti FLAG M2 antibody (Sigma-Aldrich Corporation).

### 2.4 Transmission Electron Microscopy

As has been described, zebrafish embryos were fixed in 1.5% glutaraldehyde plus 1% paraformaldehyde in 70 mM Na_2_HPO_4_ buffer pH 7.2 plus 3% sucrose at 4 °C overnight, post-stained in 1% osmium tetroxide in 0.1 M sodium cacodylate pH 7.4 buffer, dehydrated, infiltrated in a 1:1 solution of propylene oxide and EPON (Ted Pella, Inc.) and embedded in EPON (Kramer-Zucker et al., 2005). Thin sections (70–80 nm) were cut on a Reichert Ultracut E ultra-microtome and collected onto formvar-coated slot grids. The sections were stained with uranyl acetate and lead and examined in a Philips CM10 TEM at 80 kV.

### 2.5 Fluorescent Dextran Injection

1% FITC-dextran (500 kD, Molecular Probes) dissolved in PBS was injected into the common cardinal vein of 96 hpf larvae anesthetized with 0.2 mg/ml tricaine (3-amino benzoic acid ethyl ester, Sigma) in egg water (Arif et al., 2013). After overnight incubation, the embryos were fixed and sectioned. Uptake of filtered fluorescent dextran by tubular cells was evaluated in serial sections using a Nikon fluorescent microscope.

### 2.6 Rho Activation Assay

Using a Rho activation assay kit (Upstate Biotechnology), 50 embryos at 72 hpf of each group were homogenized on ice in Mg^2 +^ Lysis Buffer supplemented with Phosphatase Inhibitor Cocktail and complete protease inhibitor cocktail (Roche LTD), spin down at 14,000 rpm at 4 °C for 10 min (Sun et al., 2011). The supernatants were collected and incubated with Rhotekin RBD conjugated agarose at 4 °C for 15 min. The Rhotekin RBD-binding GTP Rho was measured by western blotting using mouse-anti-Rho A, B and C antibody (EMD Millipore, MA).

### 2.7 Quantification of Membrane-associated Dia

As described previously, 50 embryos at 72 hpf of each group were homogenized in cold homogenization buffer (10 mM Tris–HCl, pH 7.5; 1 mM EGTA; 1 mM MgCl_2_) supplemented with complete protease inhibitor cocktail (Roche LTD) (Sun et al., 2013). The lysates were centrifuged at 50,000 g and 4 °C for 60 min to pellet membrane fractions in a Beckman Optima TLX ultracentrifuge with TLA45 rotor (Beckman Coulter, Inc.). The pellets were dissolved in 1% SDS in buffer A (50 mM Tris–HCl, pH 7.5; 140 mM NaCl; 10% glycerol; 1% Triton X-100) supplemented with protease inhibitor cocktail. Dia2 protein in the whole lysates and the membrane fractions were measured by Western blotting (rabbit anti-Dia2, Santa Cruz Biotechnology, Inc). The fraction of membrane-associated Dia2 was expressed as the percentage of total Dia2.

### 2.8 CD16/CD7/NCD Crosslink Assay

A conditionally immortalized human podocyte cell line (Saleem et al., 2002) was maintained in RPMI1640 with 10% FBS, Insulin–transferrin–selenium (Gibco), and 50 IU/ml penicillin–streptomycin at 33 °C. Podocytes at permissive condition were transfected with plasmids encoding GFP-NCK1 and CD16/CD7/Nephrin cytoplasmic domain (NCD) or CD16/CD7/HA (gift of Dr. Lawrence Holzman, University of Pennsylvania). 24 h later, the cells were incubated in RPMI 1640 containing mouse anti-CD16 (Santa Cruz Biotechnology, Inc) at 37 °C for 1 h and then with in RPMI 1640 containing 1 μg/ml of Alexa Fluor594-conjugated goat anti-mouse IgG (Molecular Probes) for 1 h. Then the cells were fixed in 4% paraformaldehyde. Filamentous actin was visualized with Alexa Fluor 350-conjugated phalloidin (Molecular Probes). After mounting, the cells were observed under a confocal microscope. Podocyte expressing CD16/CD7/HA instead of CD16/CD7/NCD or podocyte without crosslink serve as two negative controls to ensure the specificity of the crosslink assay.

### 2.9 Immunohistochemical Staining

The paraffin embedded control or INF2 morphants at 72 hpf were microsectioned to a thickness of 3 μm. The transverse sections were deparaffinized, dehydrated, and followed by antigen retrieval (autoclaved for 3 min in 0.01 M citric acid buffer). The endogenous peroxidase was inactivated by 3% hydrogen peroxide. After blocking in 5% BSA in PBS, sections were incubated in rabbit anti INF2 (dilution 1:200, Sigma-Aldrich Corporation HPA000724), biotinylated anti-rabbit IgG, streptavidin HRP complex successively. Color reaction was developed by applying HRP substrate and 3,3′-Diaminobenzidine (DAB). After co-stain with hematoxylin, the sections were examined under light microscopy. For immunofluorescence staining of nephrin, after blocking in 5% BSA in PBS, sections were incubated in rabbit anti nephrin (dilution 1:200, gift of Dr. Tomoko Obara (Ichimura et al., 2013)), and then with Alexa Fluor 594-conjugated goat anti-rabbit IgG (Molecular Probes).

## 3 Results

### 3.1 INF2 Expression in Zebrafish

**Fig. 1:**
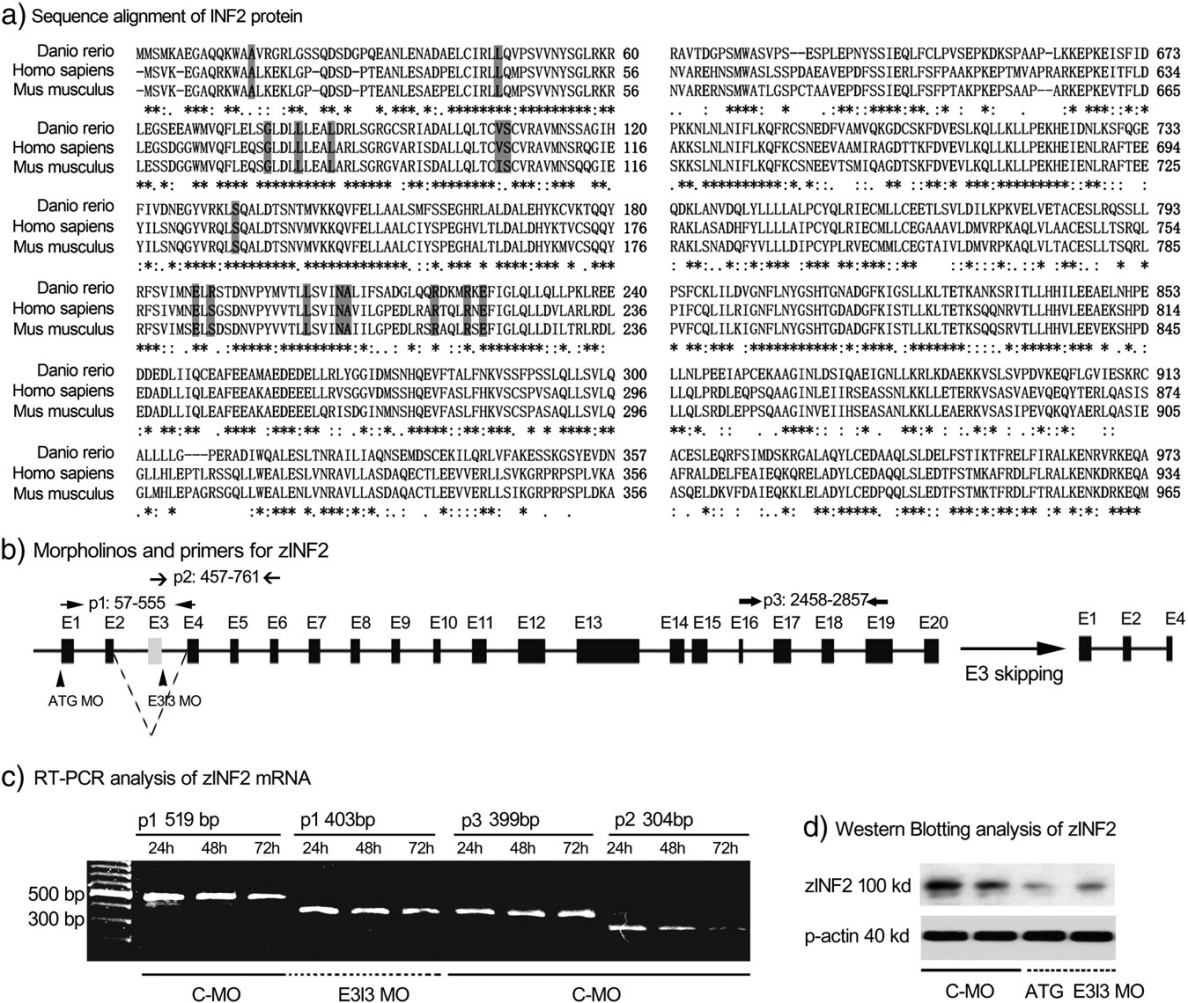
INF2 expression in zebrafish. a. Sequencing alignment of INF2 protein in zebrafish, human and mouse. The INF2 sequence similarity in zebrafish (Uniprot ID: H9GX54), human (Uniprot ID: Q27J81) and mouse (Uniprot ID: Q0GNC1) was done by using Multiple Sequence Alignment software ClustalW2. The highlighted FSGS-causing mutations occur in evolutionarily conserved residues within the DID of INF2. b. Schematic showing zINF2 transcription targeted by ATG translational blocking morpholino (ATG MO) or by exon E3I3 junction morpholino (E3I3 MO) resulting in exon 3 skipping, introducing an early stop codon and a short truncation. c. The transcription of zINF2 was measured by RT-PCR at different stages of development (24, 48, 72 hpf) by using 3 pairs of primers (p1, p2, and p3). Selective targeting of zINF2 by E3I3 morpholino was confirmed by RT-PCR using primer pair 1 flanking exon 3, which yield a shorter product (519 bp) compared to control morpholino (403 bp). d. The decreased expression of zINF2 protein in zINF2 ATG translation blocking morphants or E3I3 morphants was confirmed by western blotting.

**Fig. 2:**
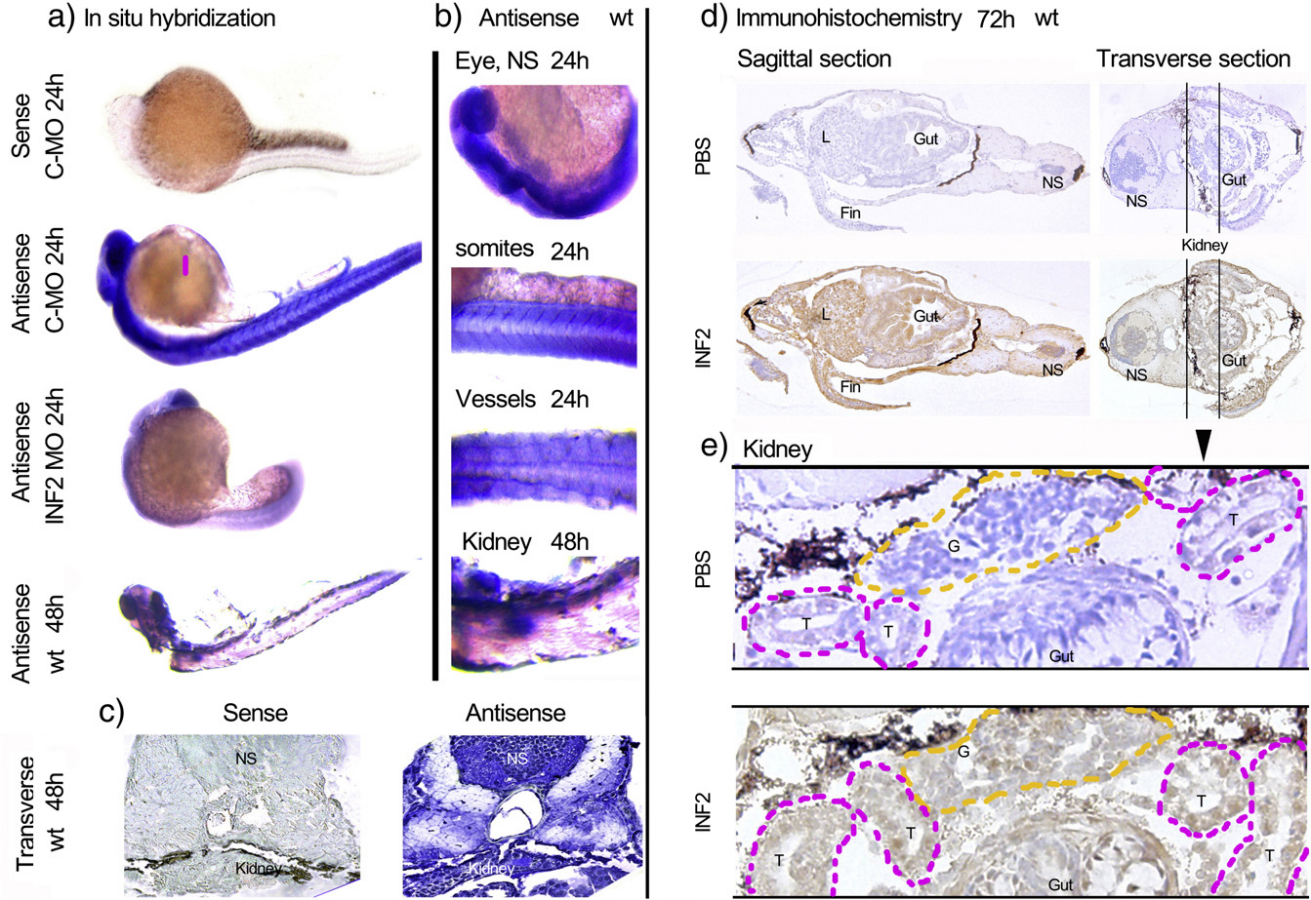
INF2 fish expression in zebrafish as detected by in situ hybridization and immunohistochemistry. a. The transcription of zINF2 at different stages of development was illustrated by using in situ hybridization (sense probe serving as a control). b. Detailed localization of INF2 transcription in wt zebrafish. c. A transverse section at kidney plane shows INF2 mRNA transcription (sense probe as a control). Immunohistochemistry stain shows INF2 expression in zebrafish at sagittal, transverse sections (d) and at kidney plane (PBS instead of 1st antibody was used in control sections). NS: nervous system; L: liver; G: Glomerulus; T: tubules.

Blast analysis of human INF2 protein sequence in the Zebrafish Model Organism Database (ZFIN) identified a single close homolog of mammalian INF2 (ENSDARG00000015102, location Zv9_scaffold3548, 66% identical at the amino acid level), in contrast to the next best matches which were less than 30% identical. The amino acid sequence similarity of these homologs in zebrafish (Uniprot ID: H9GX54), human (Uniprot ID: Q27J81) and mouse (Uniprot ID: Q0GNC1) was compared using Multiple Sequence Alignment software ClustalW2 (Fig. 1a, http://www.ebi.ac.uk/Tools/msa/clustalw2/). The transcription and the expression of zINF2 at different stages of development were determined by RT-PCR and by Western blot (Fig. 1). As shown in Fig. 2a–c, in situ hybridization shows zINF2 transcription in the nervous system (NS), eyes, somites, and vessels at the early stage of development (24 hpf), as well as significant transcription in kidney primordial at 48 hpf. Immunohistochemistry stain illustrates the expression of INF2 at nervous system, glomerulus, tubules, liver, and gut at 72 hpf (Fig. 2d and e).

### 3.2 Selective Targeting of zINF2 Causes Embryo Malformation and Impaired Nephrogenesis

A morpholino targeting the translation start ATG (ATG translational blocking MO, or ATG MO) and an alternative morpholino targeting the exon3–intron3 splice site (E313 MO) of zINF2 were used to interfere with the transcription of the gene (Fig. 1b). A standard scrambled morpholino provided by GeneTools served as a control (C-MO). This morpholino has no target or significant biological activity. Using a primer pair that flanks Exon3 (Fig. 1b), we amplified a spliced product of 400 bp in E3I3 morphants, compared to a product of 500 bp in control embryos (Fig. 1c). As indicated by sequencing of the PCR amplified products, the E3I3 MO interferes with the splicing of zINF2 mRNA, leads to exon 3 skipping, introduces an early stop codon and results in a truncated peptide (Fig. 1b). In situ hybridization (Fig. 2a) and Western blot (Fig. 1d) demonstrated decreased transcription and expression of zINF2 in INF2 morphants when compared with control embryos.

**Fig. 3:**
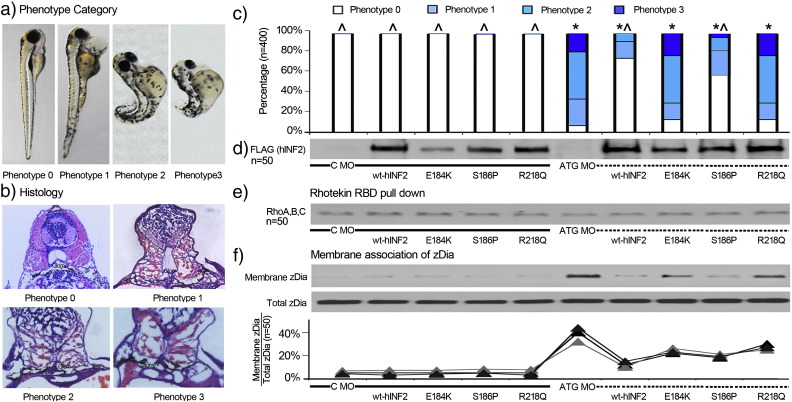
Zebrafish phenotype associated with INF2 expression and Dia activity. Control MO and INF2 ATG MO and in vitro transcribed hINF2 mRNAs were co-injected into zebrafish embryos at the single-cell stage. a, b, c. 400 zebrafish expressing different forms of INF2 (50 per group) were categorized and quantified based on different gross phenotype and histology at 96 h post fertilization (hpf). Phenotype 0: grossly and histologically identical with wt fish at 96 hpf; phenotype 1: significant pericardia edema, slightly disorganized sarcomeres at the tip of the tails, without significant tail curling or dorsalization. Histologically collapsing or underdeveloped glomerular tufts.; phenotype 2: Diffuse edema with prominent yolk, significant bending and dorsalization. Histologically, underdeveloped glomerulus without open capillary tuft.; phenotype 3: dorsalized embryo with shortened tail, diffuse edema, underdeveloped pronephros, and distended tubules. Statistical significance, *χ*^2^ test: *, *p* < 0.05 vs. C-MO only; ^, *p* < 0.05 vs. ATG-MO only. d. Expression of hINF2 protein (Flag-tagged) was confirmed by western blot in 50 zebrafish in each group. e. Rho activity in zebrafish with expression of different forms of INF2 was measured by Rhotekin RBD pulldown assay. f. Dia activity in zebrafish with different manipulations of INF2 was measured and quantified as the ratio of membranous associated Dia2 (Membrane/Total Dia2).

INF2 morphants exhibit an edematous phenotype with various degrees of dorsalization and an underdeveloped kidney on morphologic assessment (Fig. 3). We categorized each of the INF2 morphants into one of four phenotypes: 0, no significant difference vs. control; 1, pericardial edema; 2, diffuse edema with significant dorsalization; and 3, diffuse edema, more extensive dorsalization. The INF2 morphants showed markedly increased edema and glomerulosclerosis (Fig. 3).

To confirm that the morphogenetic defects in zebrafish were in fact caused by the selective targeting of INF2, we co-injected in vitro transcribed human INF2 mRNA (hINF2) together with the zINF2 ATG MO (Fig. 3). We found that the malformation of INF2 morphants could be largely rescued by the expression of wild-type hINF2, but neither E184K nor R218Q point mutant forms of hINF2 (which disrupt the INF2/Dia interaction) were able to rescue the phenotype (Sun et al., 2011, Sun et al., 2013). Embryos injected with the control morpholino (C-MO, solid line group) together with hINF2 mRNA (wild-type or mutant) did not show any significant morphogenetic defects suggesting that while these FSGS-causing INF2 mutations cause a dominantly inherited human disease, they do not act in a purely dominant gain-of-function manner (Fig. 3).

### 3.3 INF2 Knockdown in Zebrafish Causes Slit Diaphragm Deformity and Dysfunction

**Fig. 4:**
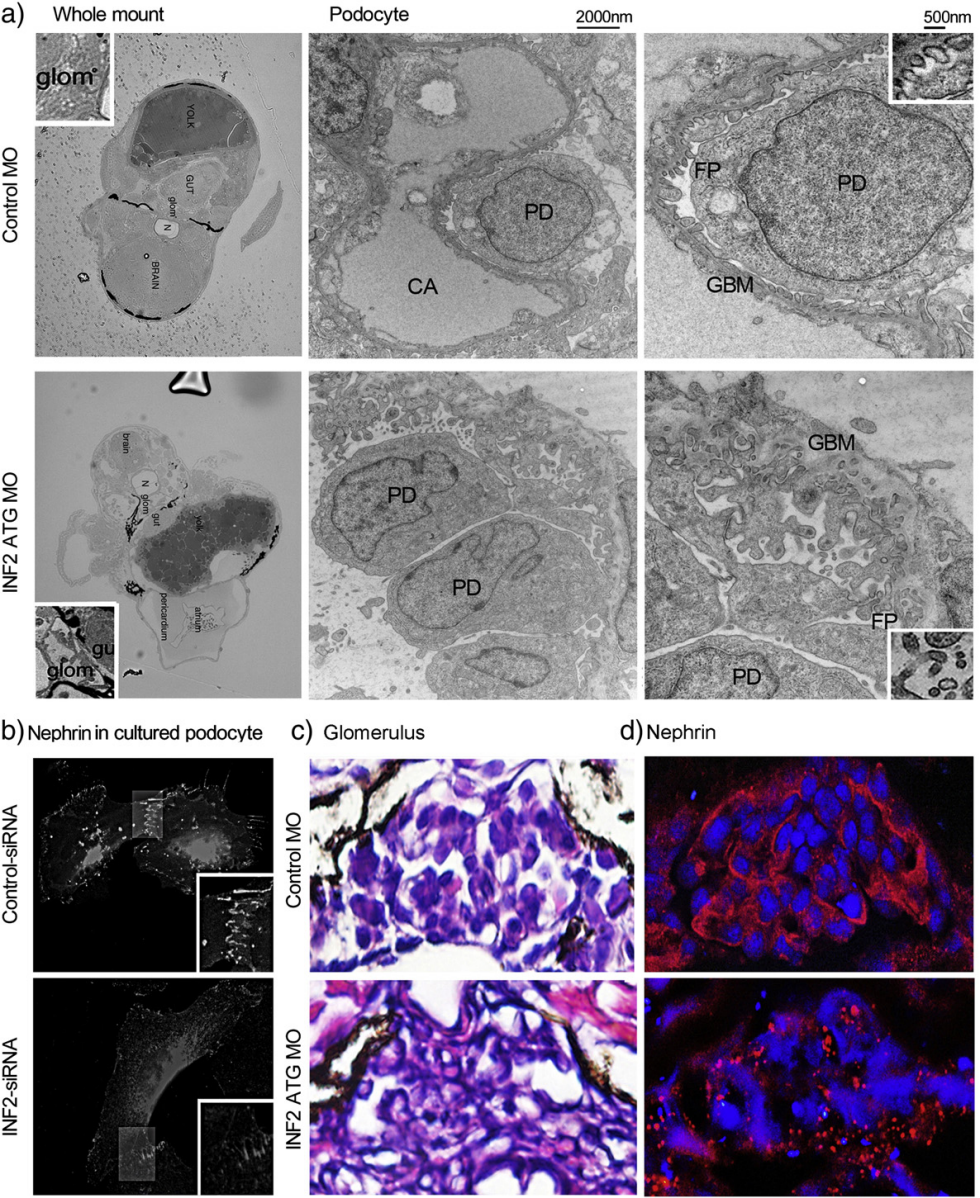
INF2 knockout leads to altered podocyte structure and nephrin mistrafficking. a. Ultrastructure of glomerulus and podocyte in INF2 morphant zebrafish vs. control fish. b. Cultured podocytes were transfected with INF2-targeting siRNA or a control RNA duplex (control siRNA) for 48 h. Then the cells in both groups were transfected with a plasmid encoding human nephrin. 24 h later, nephrin expression was illustrated by immunofluorescence stain in cultured podocyte with INF2 knockdown (INF2 siRNA) vs. control cells (control siRNA). c. Glomerular section of INF2 morphants (INF2 MO) and control fish (control MO). The expression of nephrin is illustrated using immunofluorescence staining in the zebrafish glomerulus (INF2 morphants (INF2 MO) vs. control fish (control MO)).

**Fig. 5:**
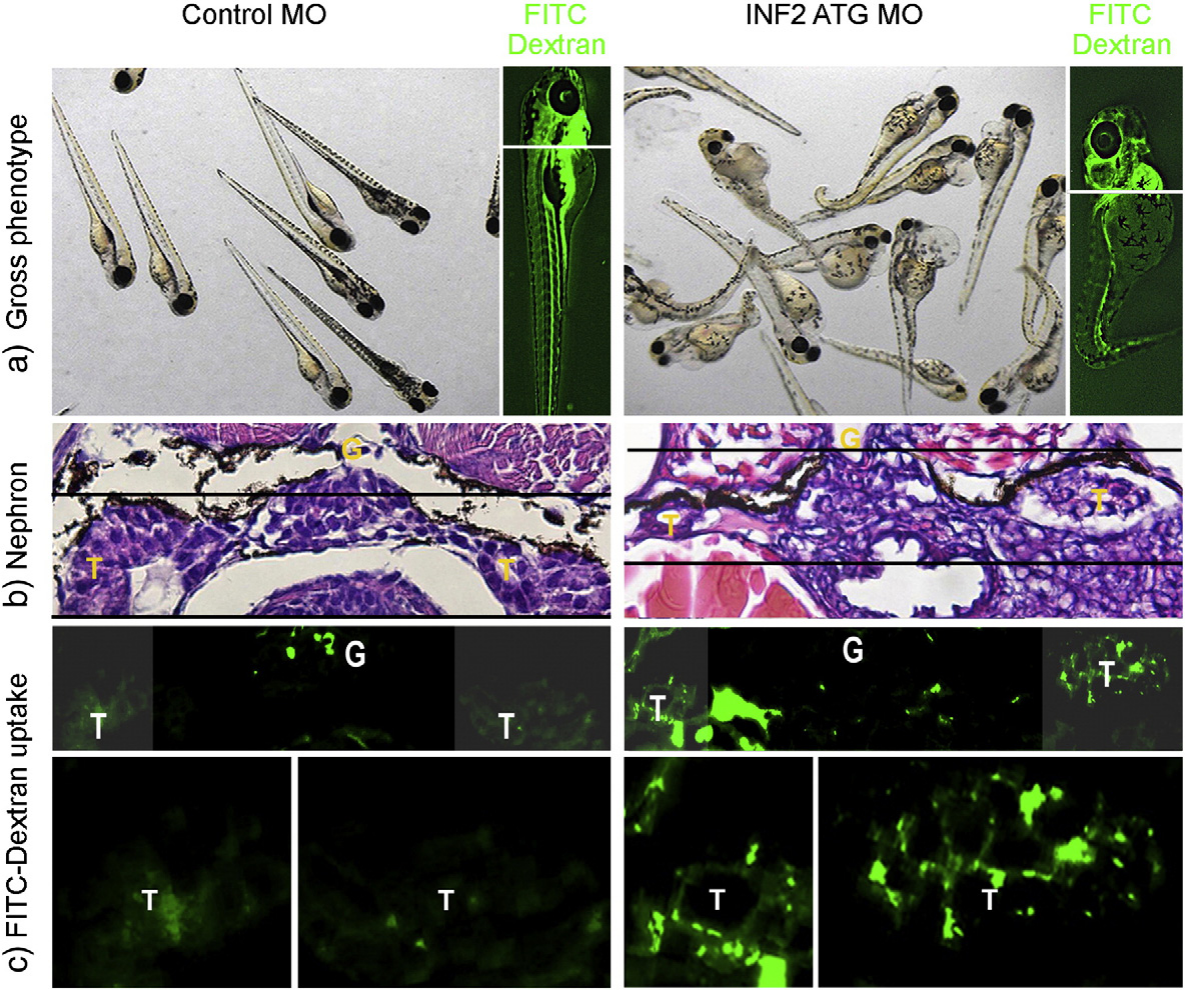
INF2 knockout in zebrafish results in slit diaphragm dysfunction. Control and INF2 morphants were examined for the effect of INF2 knockdown on the gross phenotype (a) and histology of glomerulus (G) (b). At 96 hpf, FITC-labeled dextran was injected into the cardinal vein of the fish. 24 h later, fish were sacrificed and sections at the kidney plane were examined under light microscopy (b) and fluorescence microscopy (c). Uptake of injected FITC-dextran in tubular cells indicates the increased leakage of molecules through the glomerular filtration barrier with INF knockdown (d. magnified tubular cells, INF2 MO vs. Control MO). G denotes glomerulus, T denotes tubule.

The INF2 morphants at 96 hpf develop an edematous phenotype with abnormal glomerulus and tubules (Fig. 5). Electron microscopy of the control embryos demonstrated uniform podocyte foot processes along the glomerular basement membrane (GBM). In INF2 morphants at the same developmental stage, we observed irregular cytoplasmic protrusions (i.e. jumbled foot processes) with crowded microvillus structures adjacent to an uneven GBM. In these morphant fish the intercellular and cell–matrix orientations were both disrupted, accompanied by absence of distinct slit diaphragms (Fig. 4).

To evaluate the filtration function of the glomerulus, we injected FITC labeled dextran (500 kd) into the cardinal vein of INF2 morphants or control embryos at 96 hpf (Fig. 5) (Arif et al., 2013). We observed the accumulation of FITC-dextran in the pronephric tubular cells of INF2 morphants when compared with the tubules in control embryos, indicating increased permeability of the glomerular filtration barrier in the INF2 morphants (Fig. 5).

### 3.4 INF2 Knockdown in Zebrafish Results in Mistrafficking of Slit Diaphragm Protein

The jumbled podocyte foot processes and loss of slit diaphragms in INF2 morphants suggest a role for INF2 in the maintenance of these intercellular junctions. We examined the expression of nephrin, a transmembrane protein unique to the podocyte slit diaphragm with both structural and signaling roles. In cultured human podocytes, nephrin localizes to the lamellepodial edge with enrichment at the intercellular junctions along the interdigital processes (Fig. 4), compared to the deficient nephrin trafficking in podocytes treated with INF2 siRNA. In our in vivo zebrafish model, nephrin exhibits a linear distribution along the capillary tufts in the control glomeruli compared with a patchy granular distribution of nephrin in the glomeruli of INF2morphants (Fig. 4).

### 3.5 INF2 Depletion in Podocytes Disrupts Slit Diaphragm Signaling

**Fig. 6:**
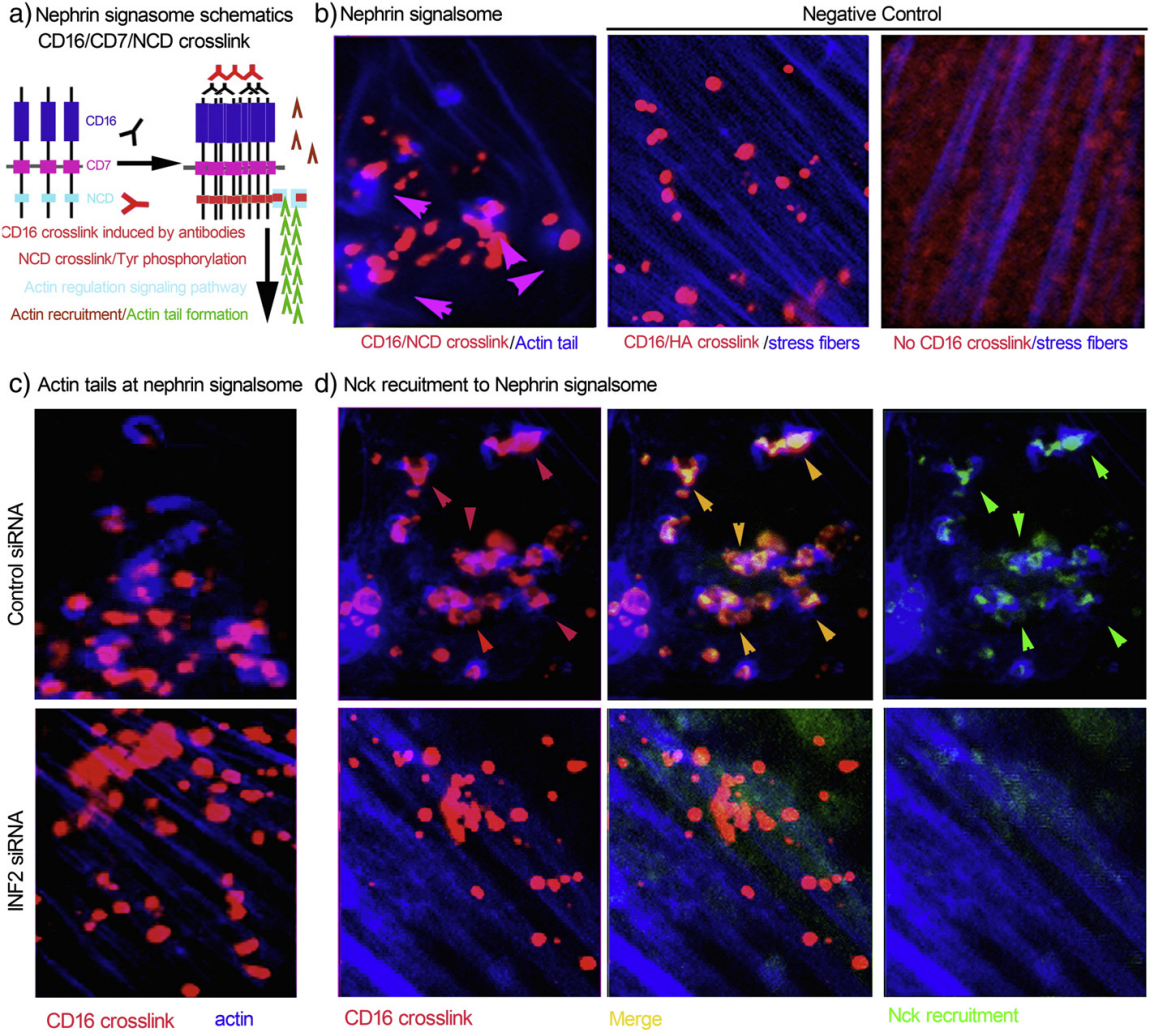
INF2 depletion in cultured podocytes destroys the integrity of slit diaphragm signaling. a. Schematic of CD16/Nephrin cytoplasmic domain (NCD) crosslink model. In cultured human podocytes expressing recombinant CD16/CD7/NCD, anti-CD16 antibody and Alexa Fluor594-conjugated secondary antibody were applied to induce CD16/NCD crosslink. The crosslink leads to phosphorylation of Tyr residuals in NCD, recruitment of Nck and actin remodeling. b. The successful crosslink exhibits blue actin tails attached to red nephrin signalsome particles. Podocytes expressing CD16/CD7/HA with CD16 crosslinked (no actin tails attaching to CD16/HA crosslink particles) and podocytes expressing CD16/CD7/NCD without CD16 crosslinked served as two negative controls. c. In podocytes expressing CD16/CD7/NCD, INF2 knockdown disrupts GFP-Nck recruitment (d) the actin tail formation (c and d) despite successful crosslinking, leading to loss of normal of slit diaphragm signaling. (For interpretation of the references to color in this figure legend, the reader is referred to the web version of this article.)

Nephrin pairing at the slit diaphragm initiates the phosphorylation of nephrin's cytoplasmic domain, which recruits additional signaling molecules (for example, Nck1 and 2) that then induce actin remodeling. We used an established antibody-induced nephrin cytoplasmic domain (NCD) crosslinking assay (Fig. 6a) to investigate the role of INF2 in the integrity of the slit diaphragm signaling (Verma et al., 2006). CD16/NCD crosslinking results in the formation of a so-called nephrin signalsome (red) characterized by GFP-Nck (green) recruitment and actin tail formation (blue tails marked by arrows, Fig. 6b and d). As negative controls, we used either podocytes without crosslinking or podocytes expressing CD16/CD7/HA rather than CD16/CD7/NCD. No actin tails form in either of these control conditions (Fig. 6b). Knockdown of INF2 led to the disruption of normal nephrin signaling characterized by both defective Nck recruitment and loss of actin tail formation at the clustered nephrin (Fig. 6c and d).

### 3.6 INF2 Antagonizes Rho/Dia Signaling During Zebrafish Development

As shown in Fig. 3, the membrane association of zDia (indicating the activation of Dia) was significantly increased in INF2 morphants compared to the amount of membrane zDia seen in control embryos, despite similar levels of Rho activity as measured by a Rhotekin RBD pulldown assay (Fig. 3e). The co-expression of wild-type hINF2 largely corrected the phenotype of INF2 morphants and restored the uncontrolled Dia activity in comparison to the sustained Dia activation seen in INF2 morphants expressing E184K or R218Q forms of hINF2, two FSGS-causing mutations that disrupt the inhibitory effects of INF2 on Dia.

**Fig. 7:**
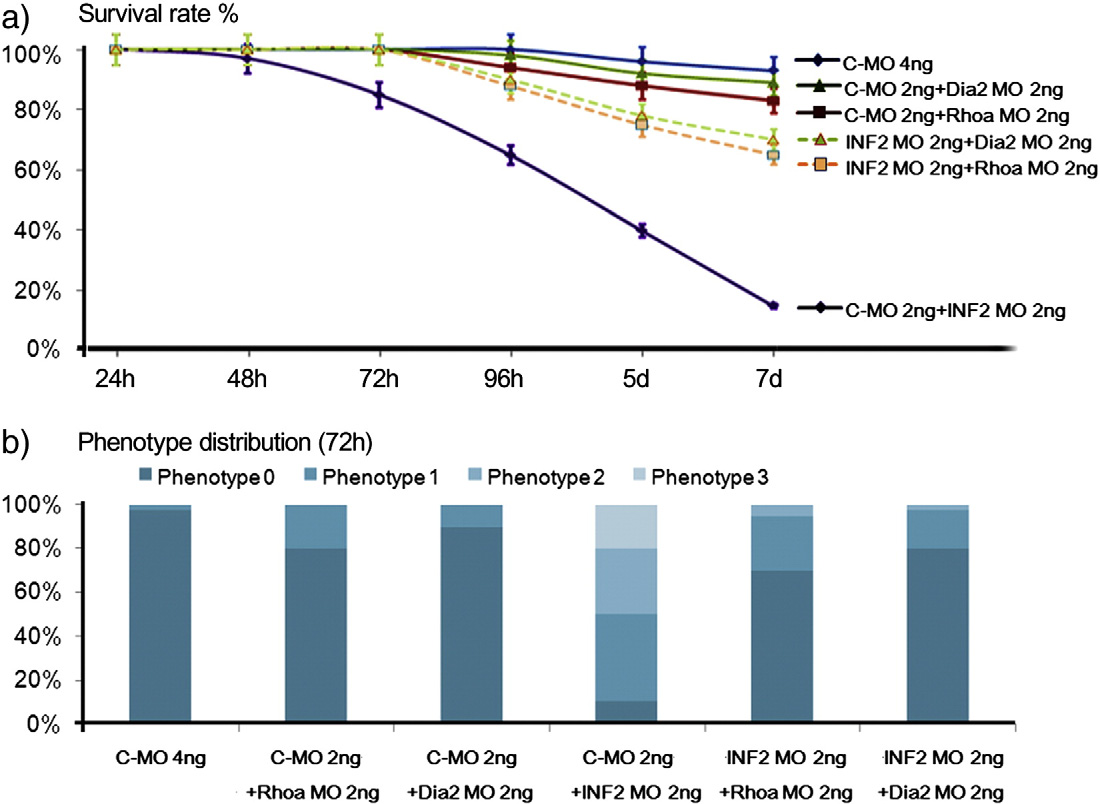
Co-injection of Rhoa or Dia2 morpholinos rescues the INF2 morphant phenotype. 100 surviving zebrafish embryos in each group (control MO, INF2 MO, Rhoa MO, INF2 + Rhoa MO, Dia MO, INF2 + Dia MO) were randomly selected. The survival rate (a) and the phenotype distribution (b; phenotype 0, 1, 2, 3 as defined above) were recorded at different stages of development (48 h, 72 h, 96 h, 5 d, and 7 d). The data was collected in 3 independent experiments. Distribution of the gross zebrafish phenotypes at 72 hpf, as defined in Fig. 3.

We next co-injected INF2 ATG MO with a morpholino designed to inactivate either zebrafish RhoA (Zhu et al., 2008) (MO4-Rhoab sequence from http://zfin.org/ZDB-MRPHLNO-080923-1) or zebrafish Dia2 MO (Lai et al., 2008) (DIAPH2-001, ENSDART00000147443, ATG translational blocking MO sequence from http://zfin.org/ZDB-MRPHLNO-090115-1), in order to confirm that the alterations seen in INF2 morphants are a result of INF2's role in opposing Rho/Dia signaling. Subthreshold doses of Rhoa MO (2 ng/embryo) or Dia2 MO (2 ng/embryo), either of which alone does not induce a significant phenotype, was co-injected with INF2 ATG MO (2 ng/embryo) or control MO (2 ng/embryo). The survival rates and phenotype distributions were compared at 72 hpf (Fig. 7). Co-injection of Rhoa or Dia2 largely rescued the abnormal morphogenesis phenotypes and reduced survival induced by INF2 morpholinos.

## 4 Discussion

Our analysis of an INF2 deficient zebrafish model shows that INF2 is essential for normal embryonic development in zebrafish, and specifically for the development and maintenance of the integrity of the glomerular filtration barrier. Loss of INF2 function leads to a nephrosis-like edematous phenotype with podocyte abnormalities accompanied by both malformation and malfunction of the glomerular filtration barrier.

The podocyte, a highly differentiated glomerular epithelial cell, makes up the most distal portion of the kidney filtration barrier. Recent studies show that Rho GTPase plays a key role in maintaining podocyte morphology and function (Wang et al., 2012). Excess activation of RhoA in podocytes induces proteinuria and FSGS (Zhu et al., 2011). Our earlier studies in cultured podocytes demonstrated that INF2 modulates Rho/Dia signaling by binding to and antagonizing the actin polymerizing activity of Dia, a major downstream effecter of RhoA. Here we demonstrate that the activity of Dia, as measured by its membrane association, correlates with the severity of the observed INF2-deficient zebrafish phenotype, despite the absence of significant Rho activation. These data suggest that INF2 likely functions though the direct opposition of the Dia protein, rather than through activation of Rho.

Ultrastructurally, the podocytes of INF2 morphants lose the normal regularity of podocyte foot processes and normal slit diaphragm structure. Instead, they exhibit jumbled protrusions with prominent microvillus formation and loss of normally oriented slit diaphragms. Microvilli are finger-like membrane protrusions with central actin bundle elongation characterized by Dias localized to the tip of these protrusions and consistent with excess Rho/Dia signaling in these INF2 morphants (Ridley, 2006). Trafficking of nephrin to specific membrane domains of precursor podocytes during development has been shown to initiate foot process budding and intracellular junction formation (slit diaphragms) (George and Holzman, 2012). Mistrafficking of slit diaphragm proteins may result from unbalanced Rho/Dia signaling in podocytes with INF2 deficiency (Sun et al., 2013, Homem and Peifer, 2009, Wallar et al., 2007). In the present studies, we found a significant change in nephrin trafficking in INF2 morphant zebrafish. These findings suggest that uncontrolled Dia activity in the absence of INF2 contributes to the abnormal development of the glomerular filtration barrier, caused at least in part by defective trafficking of slit diaphragm proteins.

In addition to the mistrafficking of nephrin in cultured podocytes with INF2 knockdown and in INF2 morphant zebrafish, we also found loss of the normal signaling pattern from the slit diaphragm complex that is normally initiated by nephrin crosslinking. Defective recruitment of signaling components and loss of the usual actin tail formation at the nephrin signalosome were observed in INF2 deficient podocytes, indicating a critical role for INF2 in maintaining the function of the slit diaphragm and glomerular filtration barrier.

The defective phenotype of these zINF2 morphants was efficiently rescued by the wild-type hINF2 but not by disease-causing mutant forms E184K, R218Q. This finding is consistent with the model that human disease-causing mutations located within the DID of INF2 act, at least in part, via the loss of INF2-mediated dampening of Rho/Dia activity. We observed that knockdown of either RhoA or Dia2 was sufficient rescue the zINF2 morphant phenotype. This supports the idea that the pathologic effects of INF2 loss of function are mediated, at least in part, by loss of inhibition of Rho/Dia signaling.

We used morpholinos rather than gene editing technologies to explore the relationship between INF2 dose and the final output of the in vivo assays. While there may sometimes be issues with the specificity of morpholinos, these are allayed in this case by the precision of the final phenotype, the initial titration dose response curves and the effective rescue by wildtype human INF2. Subsequent experiments were performed using doses of morpholino that were well below those at which any potential non-specific phenotypes were observed and where there was complete rescue with wild type INF2 RNA.

In summary, our studies demonstrate that INF2 dampens Rho/Dia mediated actin dynamics and actin-dependent behaviors in vivo. By means of this activity, INF2 preserves the oriented trafficking of glomerular slit diaphragm proteins and the integrity of signaling at the slit diaphragm complex. Without the counterbalancing effects of INF2, Rho/Dia signaling is not restrained, resulting in both structural and functional abnormalities of the podocyte as well as failure to establish a definitive glomerular filtration barrier. These findings help elucidate the regulatory mechanisms governing Rho activity in general and Rho-regulated glomerular function in particular. These results also add to a growing body of literature regarding the complexity and regulatory interactions governing small GTPases, cytoskeletal regulation and their relevance to human disease. Given that podocyte dysfunction is a common feature of most proteinuric forms of kidney disease, understanding the mechanisms of podocyte injury, and how the effectors of mechanisms might be modulated for therapeutic benefit, we believe that these experiments provide new clues as to how Rho-mediated injury might be modulated.

The simple zebrafish model that we employ here should be useful for both the further dissection of the biological pathways involved in INF2 mediated human glomerular disease, and perhaps INF2 mediated neurological disease as well. The unique characteristics of zebrafish should also make this a useful model for the identification of pharmacologic agents that modify these diseases.
